# *Src* Mutation Induces Acquired Lapatinib Resistance in *ERBB2*-Amplified Human Gastroesophageal Adenocarcinoma Models

**DOI:** 10.1371/journal.pone.0109440

**Published:** 2014-10-28

**Authors:** Yong Sang Hong, Jihun Kim, Eirini Pectasides, Cameron Fox, Seung-Woo Hong, Qiuping Ma, Gabrielle S. Wong, Shouyong Peng, Matthew D. Stachler, Aaron R. Thorner, Paul Van Hummelen, Adam J. Bass

**Affiliations:** 1 Department of Medical Oncology, Dana-Farber Cancer Institute, Boston, Massachusetts, United States of America; 2 Department of Oncology, Asan Medical Center, University of Ulsan College of Medicine, Seoul, Korea; 3 Department of Pathology, Asan Medical Center, University of Ulsan College of Medicine, Seoul, Korea; 4 Division of Hematology/Oncology, Beth Israel Deaconess Medical Center, Boston, Massachusetts, United States of America; 5 Innovative Cancer Research, Asan Institute for Life Science, Asan Medical Center, University of Ulsan College of Medicine, Seoul, Korea; 6 Cancer Program, The Broad Institute of MIT and Harvard, Cambridge, Massachusetts, United States of America; 7 Department of Pathology, Brigham and Women’s Hospital, Boston, Massachusetts, United States of America; 8 Center for Cancer Genome Discovery, Dana-Farber Cancer Institute, Boston, Massachusetts, United States of America; 9 Department of Medicine, Harvard Medical School, Boston, Massachusetts, United States of America; 10 Department of Medicine, Brigham and Women’s Hospital, Boston, Massachusetts, United States of America; Complutense University, Spain

## Abstract

ERBB2-directed therapy is now a routine component of therapy for *ERBB2*-amplified metastatic gastroesophageal adenocarcinomas. However, there is little knowledge of the mechanisms by which these tumors develop acquired resistance to ERBB2 inhibition. To investigate this question we sought to characterize cell line models of *ERBB2*-amplified gastroesophageal adenocarcinoma with acquired resistance to ERBB2 inhibition. We generated lapatinib-resistant (LR) subclones from an initially lapatinib-sensitive *ERBB2*-amplified esophageal adenocarcinoma cell line, OE19. We subsequently performed genomic characterization and functional analyses of resistant subclones with acquired lapatinib resistance. We identified a novel, acquired *Src* ^E527K^ mutation in a subset of LR OE19 subclones. Cells with this mutant allele harbour increased Src phosphorylation. Genetic and pharmacologic inhibition of Src resensitized these subclones to lapatinib. Biochemically, *Src* mutations could activate both the phosphatidylinositol 3-kinase and mitogen activated protein kinase pathways in the lapatinib-treated LR OE19 cells. Ectopic expression of *Src* ^E527K^ mutation also was sufficient to induce lapatinib resistance in drug-naïve cells. These results indicate that pathologic activation of Src is a potential mechanism of acquired resistance to ERBB2 inhibition in *ERBB2*-amplified gastroesophageal cancer. Although *Src* mutation has not been described in primary tumor samples, we propose that the Src hyperactivation should be investigated in the settings of acquired resistance to ERBB2 inhibition in esophageal and gastric adenocarcinoma.

## Introduction

Gastroesophageal (GE) adenocarcinomas are one of the leading causes of the cancer death worldwide [1]. The mainstay of systemic chemotherapy for patients with advanced or metastatic disease still consists of cytotoxic agents including fluoropyrimidines, platinum derivatives, taxanes and topoisomerase inhibitors [2]–[5]. However, a recent randomised trial demonstrated that trastuzumab, a humanized IgG1 monoclonal antibody targeting human epidermal growth factor receptor-2 (ERBB2 or HER2), improved overall survival by 2.7 months in patients with *ERBB2*-amplified advanced gastroesophageal cancer when combined with chemotherapy [6]. Based upon these results, trastuzumab is now a routine component of care for patients with metastatic *ERBB2*-amplified GE adenocarcinomas.

Despite the adoption of ERBB2 inhibitor therapy in clinical practice, the addition of anti-HER2 targeting strategies in patients with *ERBB2* amplified gastroesophageal cancer have been modest, attributable both to intrinsic resistance of many tumors to trastuzumab containing therapy as well as to the emergence of acquired resistance in those tumors which initially responded to treatment. The etiology of resistance to ERBB2-directed therapies has been widely investigated in breast cancer [7]–[15]; the accepted resistance mechanisms included constitutive activation of the PI3-K pathway [7], [9], truncated p95 isoform of HER2 receptor which cannot bind to trastuzumab [15], and constitutive Src activation as a common node downstream of multiple pathways [10]. In GE cancer, the variant of dopamine and cyclic AMP-regulated phosphoprotein (t-DARPP) has been suggested as a resistance mechanism to ERBB2 inhibitors [16], and exogenous HGF administration to cell line cultures has been shown to induce *in vitro* resistance in GE adenocarcinoma [17]. In one report, NCI-N87 *ERBB2*-amplified gastric adenocarcinoma cells were found to acquire enhanced activity of Src activity following prolonged in vitro exposure to trastuzumab [18]. Beyond these reports, there is little understanding the etiology of acquired or *de novo* resistance to ERBB2 inhibition in GE cancer.

To address this problem, various clinical efforts are evaluating the empiric potential of distinct second-line agents to improve survival and clinical responses [19]–[22]. To guide the development of such treatment strategies, we sought to investigate the potential mechanisms of acquired resistance to ERBB2 inhibition in *ERBB2*-amplified GE adenocarcinoma cell line models. Indeed, in other tumor types, study of means of resistance in cell line models has identified resistance mechanisms subsequently validated in primary cancers [9], [10], [14]. Although trastuzumab is utilized in clinical practice, trastuzumab has limited efficacy in *in vitro* culture compared to direct kinase inhibitors such as lapatinib [23], [24]. Therefore, we have chosen lapatinib as our tool compound to identify mechanisms by which *ERBB2*-amplified GE adenocarcinomas can bypass effective ERBB2 inhibition.

From the originally lapatinib-sensitive *ERBB2*-amplified esophageal adenocarcinoma cell, OE19, we generated several resistant subclones by prolonged exposure to lapatinib. Through genomic and functional analyses of this lapatinib-resistant model, we found that an activating mutation of *Src* was responsible for the acquired lapatinib resistance in OE19 cells. In addition, we further demonstrated that genetic or pharmacologic blockade of Src could restore ERBB2 inhibitor sensitivity in lapatinib-resistant cells. These data establish the role of oncogene *Src* as a pharmacologically tractable candidate mediator of acquired lapatinib resistance in ERBB2-positive GE adenocarcinomas.

## Materials and Methods

### Cell lines and Reagents

OE19 cells were obtained from the European Collection of Cell Cultures (ECACC), and OE33 cells were purchased from the Sigma (St. Louis, MO). OE19 is *ERBB2*-amplified gastroesophageal cancer cell line and sensitive to lapatinib, hence OE33 is *ERBB2-* and *MET*-amplified gastroesophageal cancer cell line and has intrinsic resistance to lapatinib. OE19 and OE33 were cultured in a humidified, 5% CO_2_ atmosphere at 37°C in Roswell Park Memorial Institute (RPMI-1640; GIBCO BRL, Grand Island, NY) medium supplemented with 10% fetal bovine serum. Tyrosine kinase inhibitors (lapatinib, saracatinib, and crizotinib) were purchased from the LC laboratories and were dissolved in dimethylsulfoxide (DMSO). Trastuzumab was purchased from the Department of Pharmacy at the Dana-Farber Cancer Institute.

### Generation of Lapatinib-Resistant (LR) Subclones

Working with lapatinib-sensitive *ERBB2*-amplified esophageal adenocarcinoma cell line, OE19, whose IC_50_ to lapatinib is 200 nM, we initiated the development of lapatinib resistance by culturing the cell line in the presence of progressively increasing doses of lapatinib during three months; the final concentration of lapatinib was 3 µM and some clones survived at a low density with small colonies. Following six months of culture with drug, we were able to obtain OE19 derivatives that were capable of proliferation in the presence of 3 µM of lapatinib. We could observe that several colonies had distinct cellular morphologies at this time, and the pathologist (JK) picked some colonies with distinct morphologies and named them according to the selecting orders. We subsequently expanded distinct clonal subcultures from the resistant OE19 cells and subsequently extracted DNA from seven distinct clonal populations for genomic analysis.

### Genomic DNA extraction and Targeted Exome sequencing

Genomic DNA was extracted from the parental OE19 and isolated LR subclones using DNeasy Blood & Tissue Kit (Qiagen) per manufacturer’s protocol. DNA quality was evaluated by quantification using Quant-iT Pico Green dsDNAassay Kit (Invitrogen) per manufacturer’s protocol. DNA from these resistance cell lines (and the parental OE19) were subjected to focused exon sequencing using the Oncopanel_v2 cancer gene panel at the Center for Cancer Genome Discovery at the Dana-Farber Cancer Institute. OncoPanel_v2 represents a targeted sequencing strategy to simultaneously detect mutations, translocations and copy-number variations in archived clinical tumor specimens. Targeted sequencing was achieved by designing RNA baits to capture the exons of 504 genes with relevance to cancer.

Sequencing libraries were prepared, as previously described [25], starting from 100 ng of genomic DNA. Libraries were quantified by QPCR (Kapa Biosystems, Inc., Woburn, MA) and pooled in equimolar concentrations to 500 ng total and enriched for the Oncopanel_v2 baitset using the Agilent SureSelect hybrid capture kit. The enriched targeted exon libraries were again quantified by QPCR (Kapa Biosystems, Inc., Woburn, MA) subsequently sequenced in one lane of a Hiseq2000 sequencer (Illumina Inc., San Diego, CA) in a 2× 100 bp pair-end mode. Sequence alignment, demultiplexing and variant calling, including SNV and Indels, was performed using PICARD, GATK tools, Mutect and IndeLocator as previously described [25]. Sequence results from the resistance subclones were compared to the genomic results from the parental OE19 cell line in order to identify putative somatic mutations and copy-number aberrations that are the potential etiology of resistance. Only candidate somatic alterations with mutant allele fractions >5% were considered.

### Direct DNA Sequencing Analysis

The *Src* ^E527K^ mutation (g1579a) was additionally validated by direct sequencing as follows; *Src* was PCR-amplified from genomic DNA using a 2720 thermal cycler (Applied Biosystems) using OneTaq Quick-Load 2X mix (BioLabs) with each primer (forward: 5′- GGGATGGTGAACCGCGAGGT-3′, reverse: 5′-TTCTCCCCGGGCTGGT-3′). DNA electrophoresis was performed and the pure amplified PCR product with 203 bp size was isolated using QIAquick Gel Extraction Kit (Qiagen) per manufacturer’s protocol. We performed TA cloning with purified PCR products using TOPO TA Cloning Kit, bacterial transformation and propagation with competent *E.coli*, and extracted plasmid which contained sequence target using QIAprep Miniprep Kit (Qiagen). Direct sequencing was using the M13R sequencing primer at Genewiz, Inc.

### DNA Restriction Analysis

We performed DNA restriction analysis to confirm that the *Src* ^E527K^ mutation is an acquired event owing to the prolonged lapatinib exposure, and it is not already present in the parental OE19 cells. Genomic DNA were extracted from the parental OE19 and two LR subclones harbouring *Src* mutant, and PCR-amplified as described above. Genomic DNA from the Het1A cells, a non-tumor esophageal cell line, was also tested as a negative control.

Aliquots from the PCR amplicons were digested separately with *Ban* II restriction enzyme (New England Biolabs Inc., Beverly, USA) at 37°C for 4 hours. Digestions were carried out per manufacturer’s protocol.

DNA restriction fragments were electrophoresed on 2.0% agarose gels with ethidium bromide (0.5 µg/mL). DNA fragment sizes were estimated through comparison with EZ Load 100 bp Molecular Ruler (Bio-Rad).

### Vectors and Lentiviral Infection

Lentiviruses were produced by transfecting 293T cells with FuGENE6 transfection reagent (Promega) with 300 ng of VSVG, 2.7 µg of delta-8.9 or PSPAX2 and 3 µg of each construct. Target cells were infected with each virus in the presence of polybrene (8 µg/ml) for 6 hours. Forty-two hours later, the infected target cells were selected by using a predefined concentration of puromycin (1.5 µg/ml for OE33, and 2.0 µg/ml for OE19) at least for 7 days before biological experiments.

pLKO-shSrc vectors were obtained from The RNAi Consortium, and the shRNA sequences targeting *Src* were as following; for shSrc1 (NM_198291.1, clone ID: TRCN0000038149), 5′-CCGGGACAGACCTGTCCTTCAAGAACTCGAGTTCTTGAAGGACAGGTCTGTCTTTTTG-3′; for shSrc2 (NM_198291.1, clone ID: TRCN0000038151), 5′-CCGGGTCATGAAGAAGCTGAGGCATCTCAGATGCCTCAGCTTCTTCATGACTTTTTG-3′. pLKO-shLacZ1650 served as control in the RNAi silencing experiments.

Wild-type pDONR223-Src (#23934) was purchased from Addgene Inc. (Cambridge, Massachusetts). pDONR223-Src ^E527K^ was made by site-directed mutagenesis using QuikChange II XL Site-Directed Mutagenesis Kit from Agilent Technologies, Inc. (Santa Clara, California). Primer sequences for site-directed mutagenesis (g1579a) were following; sense 5′-ACTTCACGTCCACCAAGCCCCAGTACCAG-3′ and antisense 5′-CTGGTATGGGGCTTGGTGGACGTGAAGT-3′. pLX301-Src ^wild-type^ and PLX301-Src ^E527K^ lentiviral vectors were made from pDONR223-Src ^wild-type^ and pDONR223–Src ^E527K^, respectively, by performing LR clonase reaction with pLX301 destination vector (Invitrogen). pLX301-GFP served as a control in the ectopic expression experiments.

### *In vitro* cell proliferation assay

Cells (4,000/well) were seeded in quadruplicate in 96-well plate, were treated with either vehicle or variable doses of small molecule inhibitors after 24 hours, and then were allowed to grow for 72 hours. For cells grown in trastuzumab, cells were allowed to proliferate for five days prior to assays of cell proliferation. We used Cell-titer Glo assay (Promega, Madison, Wisconsin) to measure cell viability. Percentage of inhibition of cell proliferation was calculated as [1-(treated cells/untreated cells) × 100]. The results from the cell viability assay were compared between cell lines using repeated measures analysis of variance (ANOVA) and also were tested using student *t*-test at the specific concentration. A *p* value <0.05 was considered statistically significant.

### Immunoblotting

Cells were lysed with RIPA lysis buffer (50 mM Tris-HCl pH 7.5, 150 mM NaCl, 0.1% SDS, 1% NP-40, 0.5% sodium deoxycholate) supplemented by protease inhibitor cocktail (Roche) and phosphatase inhibitor cocktails (Calbiochem). Lysates were separated on 7.5% or 8% Tris-Glycine SDS-polyacrylamide gel and were transferred to PVDF membranes (Millipore). The membranes were blocked with 5% skim milk (Bio-Rad) dissolved in TBST buffer (50 mM Tris-HCl, 150 mM NaCl, 0.05% Tween-20). Then, the membranes were incubated with primary antibodies overnight at 4°C. Anti-EGFR antibody (#A300-388A) was purchased from Bethyl Laboratories. Anti-β-actin antibody (#A5441) and anti-γ-tubulin antibody (#A9044) were purchased from Sigma-Aldrich. All other antibodies including anti-phospho EGFR Y1068 (#3777), anti-phospho ERBB2 Y1221/1222 (#2243), anti-ERBB2 (#2165), anti-phospho SRC Y416 (#6943), anti-SRC (#2109), anti-phospho-ERK 1/2 T202/Y204 (#4370), anti-ERK 1/2 (#4695), anti-phospho AKT S473 (#4060), and anti-AKT (#9272) were purchased from Cell Signaling Technologies. Horseradish peroxidase-conjugated secondary antibodies (anti-rabbit: #31460, anti-mouse: #31430, Pierce) and SuperSignal West Pico Chemiluminescent Substrate (Pierce) were used to detect signals.

## Results

### The Novel *Src* ^E527K^ mutation was found in the two lapatinib-resistant (LR) OE19 subclones

**Figure 1:**
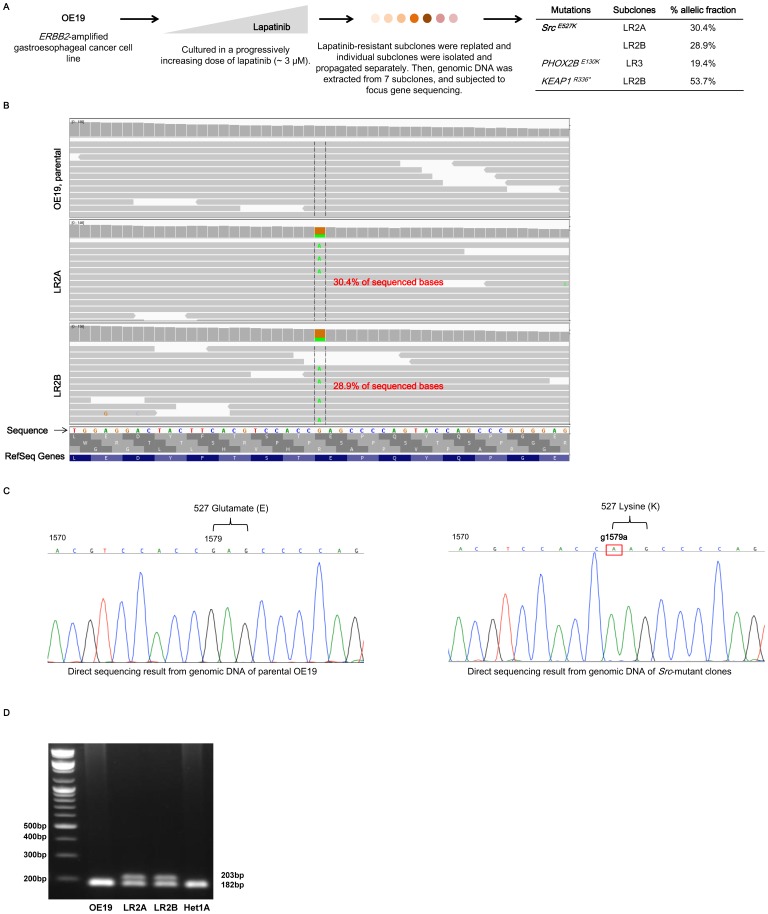
Generation of lapatinib-resistant (LR) OE19 subclones, and the identification of a novel, acquired *Src* ^E527K^ mutation. **A,** Schematic view of the development of lapatinib resistance OE19 cells followed by subcloning of distinct clones of resistant cells for genomic characterization. At right is the listing of somatic alterations identified in the distinct subclones, compared to the genome of the parental OE19 cells. The allelic fraction of each mutation, percent of sequenced reads with the mutant allele, is listed for each candidate mutation. **B,** IGV (Integrated Genomic Viewer) snapshot of *Src* mutations in two LR subclones compared to the sequencing seen from this locus in the parental, lapatinib-sensitive cell line. **C,** Direct sequencing results from genomic DNA from both parental OE19 cells and *Src* ^E527K^ mutant LR2A and LR2B subclones identifies mutation detected from next-generation sequencing. **D,** DNA restriction analysis results using *Ban* II enzyme, from the genomic DNA from the parental OE19, Src E527K mutant LR2A and LR2B subclones, and Het1A (normal esophageal cell line). Ban II enzyme cuts and yields new amplicons of 182 bp in the parental OE19 and Het1A, however, the bands of 203 bp, which contained Src mutants, are still visualized in the two Src mutant subclones, LR2A and LR2B.

For generating lapatinib-resistant subclones, OE19 cells were treated with lapatinib, of which dose was progressively increased from 200 nM to 3 µM during 3 months. We expanded the surviving clones in the presence of 3 µM of lapatinib till 6 months and colonies with distinct cellular morphologies were selected. Each selected colony was subcultured in the different plates in the presence of 3 µM of lapatinib, and 7 subclones were selected for genomic analysis (Figure 1A).

Using DNA from these seven subclones as well as from the parental OE19 cell population, we attempted to identify acquired genomic alterations that could have induced drug resistance. DNA from these distinct populations were submitted for a focused next-generation sequencing panel wherein the coding exons from 504 distinct genes were isolated via solution hybrid capture and then sequenced with an Illumina sequencer with an average depth of 252.5× and 97.1% of targets achieving a minimum coverage of 30× (Table S1). These DNA samples were analysed for the presence of somatic mutations and copy-number alterations unique to the resistance subclones compared to the parental cell line. Across these LR subclones, we identified distinct somatic mutations affecting genes *Src*, *KEAP1* and *PHOX2B* (Figure 1A). We did not find any evidence for the mutations found in the derived mutants described as above in the parental OE19 cell line of which sequencing coverage for the *Src* 527 codon of 30×.

Within these data we initially focused upon two distinct clones, both harbouring the same acquired *Src* ^E527K^ mutation (Figure 1B) which was present in ∼30% of sequenced alleles in each of these two subclones. In the setting of arm-level gain of 20q in OE19 cells, a 30% allele fraction of this mutation is consistent with one of the three copies of *Src* in the cell line being mutated clonally in this population. Notably, this specific base change, an E to K substitution at codon 527, had been utilized as a means to artificially activate Src in previous biochemical studies of this kinase [10]. Comparing the two clones with the *Src* mutation, we noted that one of the clones harboured a unique *KEAP1* mutation suggesting that these two *Src*-mutant clones may not be identical. Review of the copy-number spectrum between these two *Src*-mutant subclones, however, revealed a similar spectrum of copy-number aberrations (Figure S1) suggesting that the two clones may have diverged from a common ancestor prior to the *KEAP1* event in one subclone. Given the likely shared origin, we termed these clones lapatinib-resistant (LR) clones LR2A and LR2B. *Src* ^E527K^ mutation was confirmed by direct sequencing (Figure 1C).

Although we did not identify this *Src* mutation in the parental cell line from our sequencing, we performed additional focused analysis of this locus to detect if low-frequency *Src* mutant cells were present in the parent cell population. To identify possible mutations at low frequency, we performed restriction enzyme digestion of PCR-amplified DNA from the parental OE19, LR2A, LR2B, and Het1A using *Ban* II restriction enzyme. The target sequence of *Ban* II restriction enzyme is G(A/G)GC(T/C)C, which will cut wild-type *Src* sequence. The PCR amplicons of 203 bp from two LR subclones harboring Src mutant were not digested, in contrast those from the parental OE19 and Het1A were totally digested and yielded 182 bp products (Figure 1D). From these results, we could identify no evidence of the *Src* ^E527K^ mutant in the parental cell population, suggesting it is an acquired event during the prolonged lapatinib exposure.

### LR2A and LR2B subclones showed stable resistance to lapatinib

**Figure 2:**
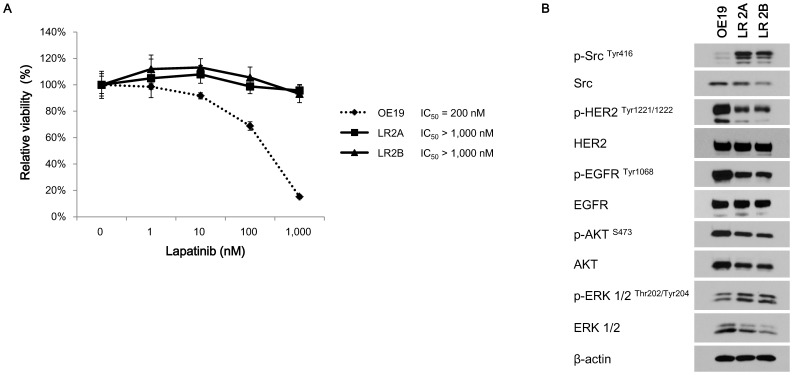
Comparisons of lapatinib sensitivities and the baseline signalling proteins activities between parental and lapatinib-resistant subclones. **A,** Lapatinib sensitivity curves in the parental OE19 and two lapatinib-resistant (LR) subclones. The calculated values of IC_50_ of lapatinib were 200 nM in parental cells and >1,000 nM in two LR subclones. Values were presented as relative cellular viability relative to vehicle-treated controls with the mean ± S.E. of quadruplicate from a representative experiment. The *p* values were <0.0001 for OE19 *vs* LR2A and OE19 *vs* LR2B, and was 0.129 for LR2A *vs* LR2B. The *p* values were calculated by two-way ANOVA. **B,** Immunoblots showing the phosphorylations of distinct signalling molecules in parental OE19 cells and the *Src*-mutant lapatinib-resistant derivatives.

With these two *Src*-mutant subclones, we re-evaluated their lapatinib sensitivity (Figure 2A). Each of these two subclones had an IC_50_ value for lapatinib greater than 1,000 nM, far exceeding that of the parental cell line. We also evaluated these two *Src*-mutant subclones for their sensitivity to trastuzumab, and they showed reduced growth inhibition to trastuzumab compared to the parental OE19 cells (Figure S2).

### Acquired *Src* ^E527K^ mutation is an activating mutation

To investigate the function of mutant *Src* within the LR2A and LR2B clones, we evaluated first Src phosphorylation at tyrosine 416, a marker of the kinase’s represent an active status [26] with immunoblotting. Both the LR2A and 2B subclones showed higher phosphorylation of Src compared to the parental OE19 cells (Figure 2B) consistent with what we would predict in the setting of an activating mutation. Additionally, in both the LR2A and LR2B clones, the expression and phosphorylation of ERBB2 and EGFR was slightly decreased relative to the parental cell line in the absence of lapatinib.

### RNAi-mediated silencing of *Src* sensitizes *Src* mutant OE19 cells to lapatinib

**Figure 3:**
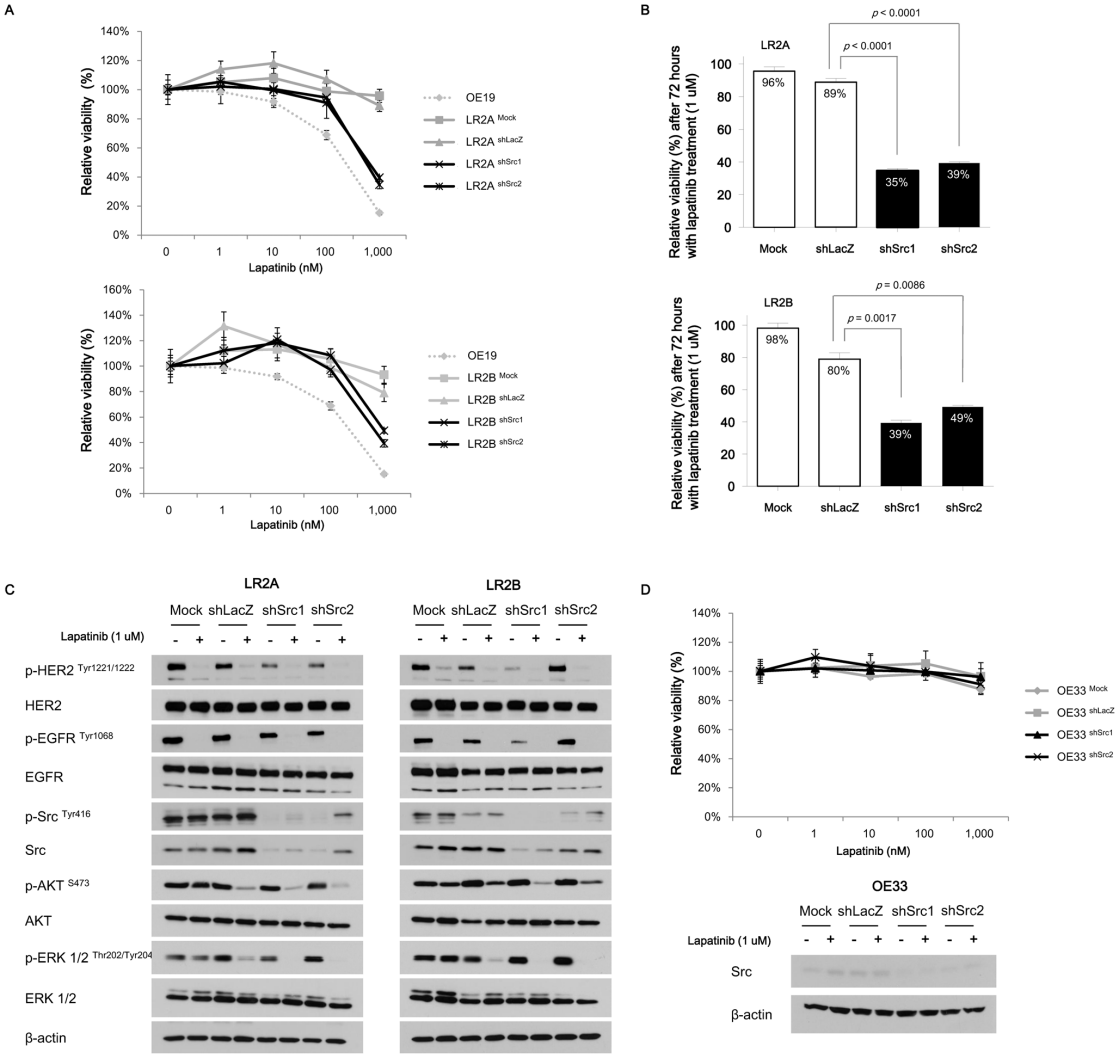
Effects of RNAi-mediated silencing of *Src* on lapatinib sensitivities and responses of signalling proteins. **A,** Lapatinib sensitivity curves for LR2A and LR2B subclones transduced with shSrc or control (shLacZ). The lapatinib sensitivities were restored after RNAi silencing of Src in the two LR subclones. The calculated values of IC_50_ were following; 200 nM for parental OE19; >1,000 nM for LR2A ^Mock^, LR2A ^shLacZ^, LR2B ^Mock^, and LR2B ^shLacZ^; 432.8 nM and 403.7 nM for LR2A ^shSrc1^ and LR2A ^shSrc2^, respectively; 276.6 nM and444.2 nM for LR2B ^shSrc1^ and LR2B ^shSrc2^, respectively. Values were presented as relative cellular viability relative to vehicle-treated controls with the mean ± S.E. of quadruplicate from a representative experiment. In the lapatinib sensitivity curve for LR2A and its subclones transduced with lentiviral vectors, the *p* value was 0.121 for mock *vs* control (shLacZ), <0.0001 for control *vs* shSrc1, and <0.0001 for control *vs* shSrc2. In the lapatinib sensitivity curve for LR2B and its subclones transduced with lentiviral vectors, the *p* value was 0.764 for mock *vs* control, <0.0001 for control *vs* shScr1, and 0.012 for control *vs* shSrc2. The *p* values were calculated by two-way ANOVA. **B,** Relative cell viability after 1 µM concentration of lapatinib treatment in two LR subclones with or without RNAi-mediated silencing of *Src*. The *p* values were calculated by two-tailed *t*-test. LR subclones transduced with shSRC restored lapatinib sensitivities. Values were presented as relative cellular viability relative to vehicle-treated controls with the mean ± S.E. of quadruplicate from a representative experiment. **C,** Immunoblots showing changes of various signalling proteins after treatment with 1 µM concentration of lapatinib in two LR subclones with or without RNAi-mediated silencing of *Src*. Proteins were harvested 4 hours after lapatinib or vehicle treatment. **D,** Lapatinib sensitivity in OE33 cells following regarding RNAi transduction targeting *Src*. Values were presented as relative cellular viability relative to vehicle-treated controls with the mean ± S.E. of quadruplicate from a representative experiment. Proteins were harvested 4 hours after lapatinib or vehicle treatment. There was no statistical significance between cell lines in terms of lapatinib sensitivity (0.070 for mock *vs* control [shLacZ], 0.520 for control *vs* shSrc1, and 0.753 for control *vs* shSrc2, respectively). The *p* values were calculated by two-way ANOVA.

Given the clear association between *Src* mutation and lapatinib resistance, we asked whether shRNA-mediated silencing of Src might restore lapatinib sensitivity in LR2A and LR2B clones. Indeed, silencing of *Src* by two independent small hairpin constructs sensitized both LR2A and LR2B clones to lapatinib treatment to the extent that the sensitivity profile of those two LR subclones became similar to that of parental OE19 (Figure 3A). Mock or control hairpin transduction did not impact the lapatinib sensitivity of all cell lines (Figure 3A–B).

Consistent with lapatinib resistance profile, we observed sustained p-ERK 1/2 phosphorylation even in the presence of 1 µM lapatinib in mock- or control hairpin-transduced LR2A and LR2B subclones. Notably, the p-ERK 1/2 phosphorylation was successfully blocked by lapatinib treatment in shSrc transduced LR subclones (Figure 3C). These data indicate that, in these *Src-*mutant LR subclones, blockade of both Src and ERBB2 is required to completely block pathologic mitogenic signalling. The phosphorylations of AKT and ERK were slightly downregulated in the shLacZ transduced LR subclones, which were not observed dominantly in the mock-treated LR subclones, possibly attributable to the effects from lentiviral infections and puromycin selection. As an additional control to ensure that these *Src*-directed shRNA vectors did not impact lapatinib sensitivity due to non-specific off target effects, we also evaluated the impact of these constructs on the lapatinib sensitivity of OE33 cell line which is not sensitive to lapatinib through unrelated mechanism, *MET* co-amplification. In this model, introduction of the sh*Src* did not sensitize OE33 cells to lapatinib (Figure 3D).

### Lapatinib resistance could be overcome by combination treatment with saracatinib

We then tested whether pharmacologic inhibition of Src by saracatinib in LR2A and LR2B subclones could restore lapatinib sensitivity. Saracatinib is a dual tyrosine kinase inhibitor targeting c-Src/Abl kinase. Saracatinib showed preclinical activity in various cancer cell lines including gastroesophageal cancers [27]–[34]; however, only low to modest antitumor activity were demonstrated in several phase I or II clinical trials when tested as monotherapy [35]–[38].

**Figure 4:**
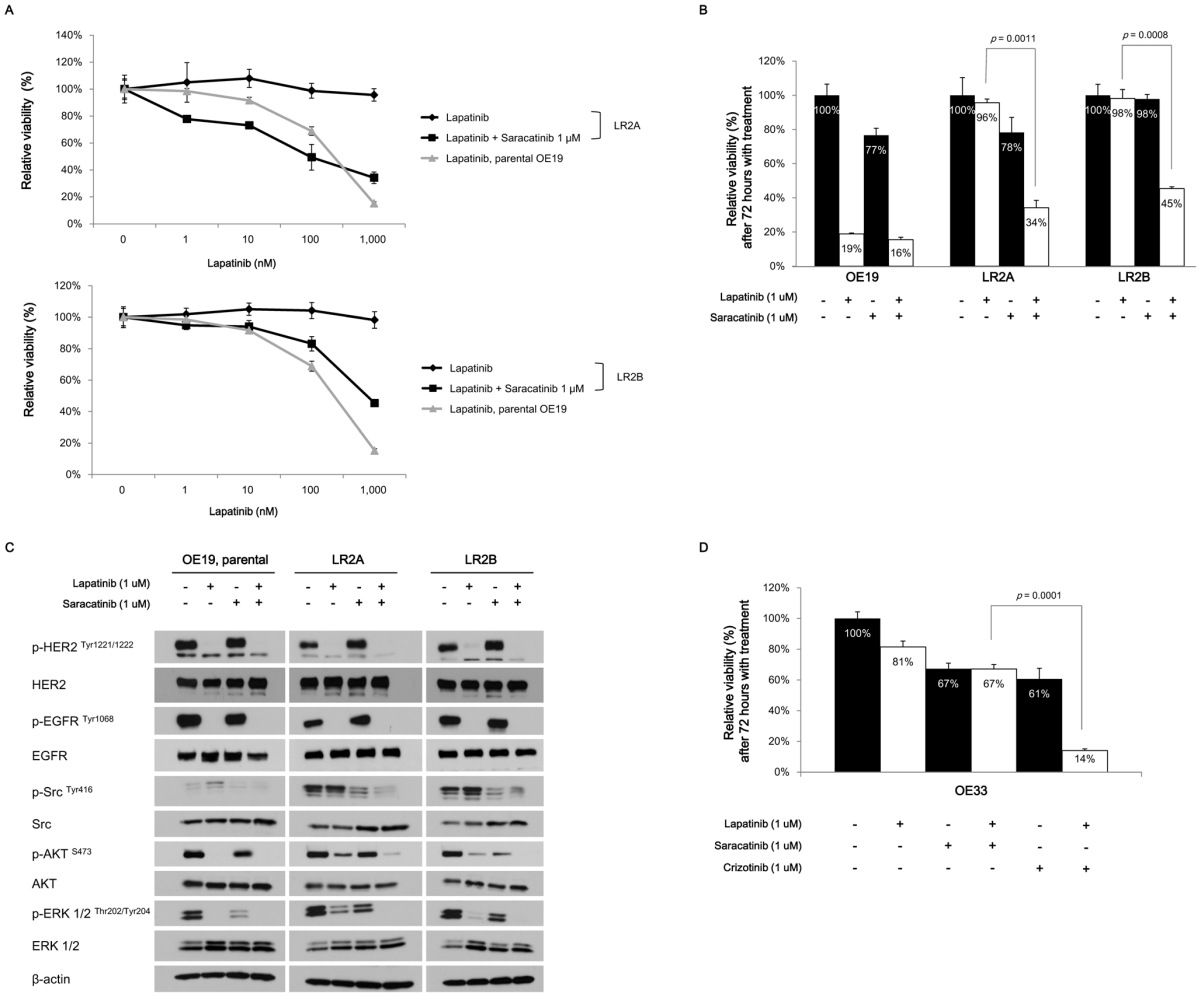
Effects of pharmacologic inhibition of *Src* using saracatinib in combination with lapatinib in LR subclones. **A,** Dose-response curves from escalated dose of lapatinib in the presence of 1 µM concentration of saracatinib. The calculated values of IC_50_ for lapatinib were following; 207.3 nM and 145 nM, respectively, after lapatinib alone and in the presence of saracatinib for parental OE19; >1,000 nM after lapatinib alone for LR2A and LR2B; 91.66 nM and 224.0 nM in the presence of saracatinib in LR2A and LR2B, respectively. Values were presented as relative cellular viability relative to vehicle-treated controls with the mean ± S.E. of quadruplicate from a representative experiment. The *p* values calculated by two-way ANOVA were <0.0001 comparing lapatinib alone with lapatinib plus saracatinib both in the LR2A and LR2B. **B,** Relative cell viability in the parental OE19 and two LR subclones after 1 µM concentration of saracatinib or lapatinib, either alone or combination. The *p* values were calculated by two-tailed *t*-test. Values were presented as relative cellular viability relative to vehicle-treated controls with the mean ± S.E. of quadruplicate from a representative experiment. **C,** Immunoblots showing changes of in intracellular signalling proteins after treatment with 1 µM concentration of lapatinib or saracatinib, either alone or combination, in LR subclones. Proteins were harvested 4 hours after each treatment. **D,** Relative cell viability in the OE33, *ERBB2* and *MET* co-amplified gastroesophageal cancer cells, after treatment with various drugs either alone or combination. The effect of combination treatment with lapatinib and saracatinib did not differ either from lapatinib alone or saracatinib alone; the combination treatment with lapatinib and crizotinib, which is a potent MET inhibitor, showed synergistic effects. The *p* values were calculated by two-tailed *t*-test. Values were presented as relative cellular viability relative to vehicle-treated controls with the mean ± S.E. of quadruplicate from a representative experiment.

While the two *Src* ^E527K^ mutant subclones were not sensitive to either lapatinib or saracatinib alone, their growth was effectively inhibited when the two drugs were combined (Figure 4A & 4B). Consistent with this drug sensitivity profile, AKT and ERK phosphorylation was sustained when the LR subclones were treated with either lapatinib or saracatinib alone, and their sustained phosphorylation was blocked upon the combined treatment of both drugs (Figure 4C). HER2 and EGFR phosphorylations were blocked upon lapatinib treatment regardless of the presence of saracatinib in both the parental OE19 cells and the two LR subclones suggesting that the mutant *Src*-mediated resistance is independent of HER2 or EGFR signalling (Figure 4C). These data suggest that pathologic Src activation enhances survival of lapatinib resistant clones upon ERBB2 inhibition through activation of both PI3-K and MAPK pathways. Saracatinib alone could not inhibit cellular proliferation either in the parental or in the two LR subclones (Figure S3). In *MET* co-amplified OE33 cells, saracatinib treatment did not show any synergy with lapatinib treatment. By contrast, the addition of MET inhibitor crizotinib synergistically inhibited cell proliferation (Figure 4D).

### Ectopic expression of *Src* ^E527K^ induces lapatinib resistance in the parental OE19 cells

**Figure 5:**
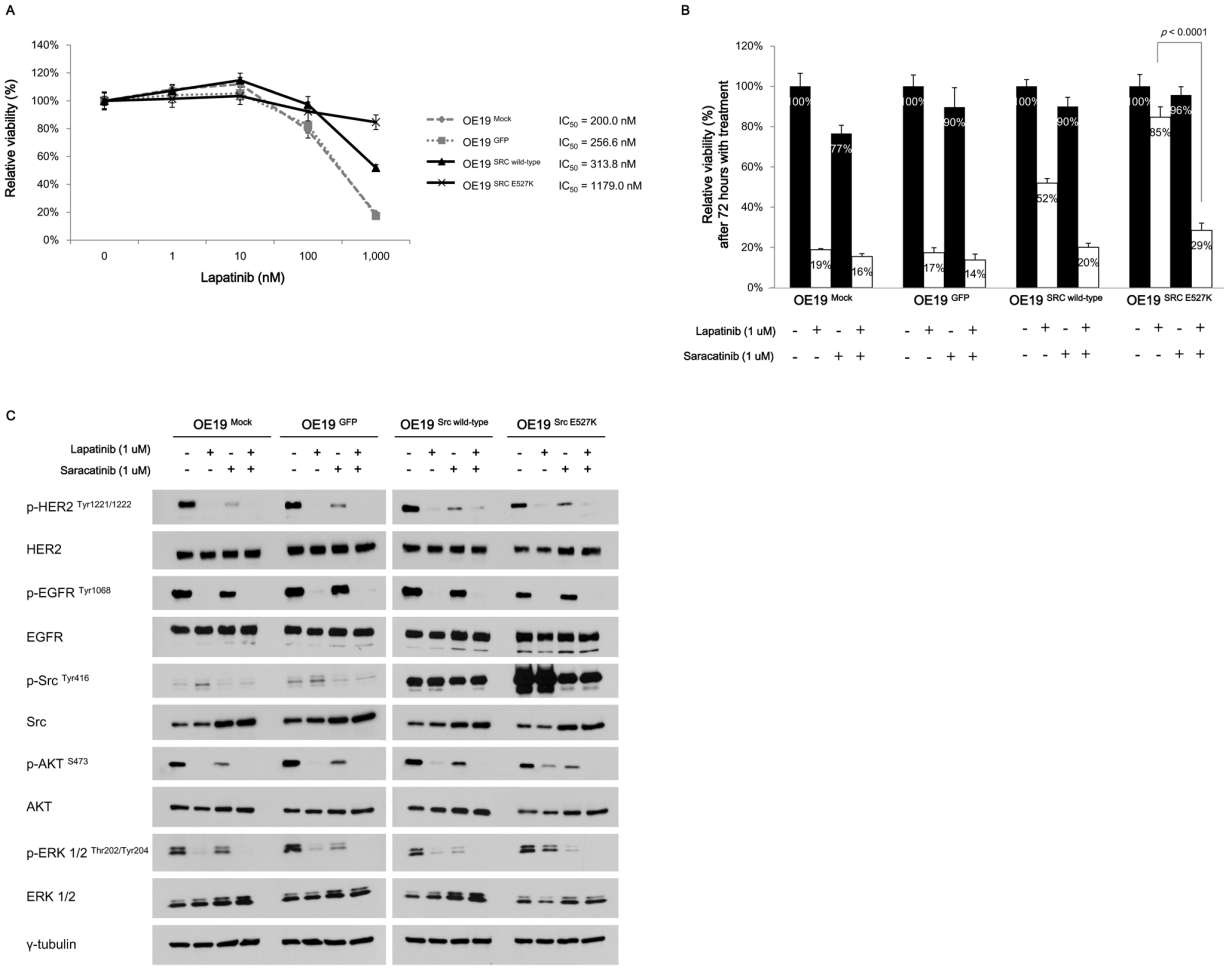
Impact of ectopic expression of *Src* ^E527K^ in parental OE19 on lapatinib sensitivity and cell signalling. **A,** Dose-response curves for lapatinib in the non-transduced parental OE19 (OE19 ^Mock^), transduced with GFP (OE19 ^GFP^), wild-type *Src* (OE19 ^Src wild-type^) or *Src* ^E527K^ mutation (OE19 ^Src E527K^). The calculated values of IC_50_ for lapatinib was >1,000 nM in OE19 ^Src E527K^ cells. Values were presented as relative cellular viability relative to vehicle-treated controls with the mean ± S.E. of quadruplicate from a representative experiment. The *p* value calculated by two-way ANOVA was 0.376 for mock *vs* control (GFP), <0.0001 for control *vs* wild-type Src, and <0.0001 for control *vs Src* ^E527K^. **B,** Relative cell viability lapatinib in the non-transduced parental OE19 (OE19 ^Mock^), transduced with GFP (OE19 ^GFP^), wild-type *Src* (OE19 ^Src wild-type^) or *Src* ^E527K^ mutation (OE19 ^Src E527K^). The *p* values were calculated by two-tailed *t*-test. Values were presented as relative cellular viability relative to vehicle-treated controls with the mean ± S.E. of quadruplicate from a representative experiment. **C,** Immunoblots showing changes of various signalling proteins after treatment with 1 µM concentration of lapatinib or saracatinib, either alone or combination, in the OE19 cells with or without Src ^E527K^ transduction.

Last we evaluated the ability of exogenous expression of the *Src*-mutants to impact the lapatinib sensitivity of OE19 cells. We generated the *Src* ^E527K^ plasmid using site-directed mutagenesis, and transduced either wild-type *Src*, mutant *Src* or GFP control into parental OE19 cells. Indeed, *Src* ^E527K^ transduced OE19 developed lapatinib resistance (IC_50_ to the lapatinib, 1179.0 nM), while GFP or *Src* ^wild-type^ transduced OE19 remained sensitive to lapatinib (IC_50_ to the lapatinib, 256.6 nM and 313.8 nM, respectively, Figure 5A). Furthermore, the lapatinib resistance conferred by exogenous expression of *Src* ^E527K^ was overcome by the combined treatment of saracatinib (Figure 5B). Saracatinib also reversed the mild lapatinib resistance induced by the wild-type *Src* transduction.

As in the LR subclones harbouring spontaneous *Src* mutation, expression of p-Src was increased in *Src* transduced OE19 either with wild-type or E527K mutant compared to the parental or GFP transduced cells (Figure 5C). *Src* ^E527K^ transduced OE19 showed particularly high expression of p-SRC. Although the phosphorylation of Src in the OE19 cells transduced with wild-type Src was not effectively inhibited by saracatinib, Src phosphorylation in *Src* ^E527K^ transduced OE19 was significantly inhibited by saracatinib regardless of the presence of lapatinib. Additionally, the levels of p-AKT and p-ERK 1/2 were not suppressed by lapatinib alone in the *Src* ^E527K^ transduced OE19, consistent with our hypothesis that constitutive Src activation might sustain both downstream signalling pathways. However, phosphorylation of both AKT and ERK 1/2 was inhibited with combination of saracatinib and lapatinib, paralleling the results from cells with spontaneously acquired *Src* mutant. Taken together, pathologic Src activation could induce lapatinib resistance in *ERBB2*-amplified GE adenocarcinoma and the resistance could be reversed by the additional Src inhibition.

## Discussion

As an effort to model acquired resistance of *ERBB2*-amplified GE adenocarcinomas to ERBB2 inhibition, we generated lapatinib-resistant subclones from an initially lapatinib-sensitive *ERBB2*-amplified esophageal adenocarcinoma cell line by prolonged exposure to the inhibitor. Through genomic and functional analysis of LR subclones, we found that an activating mutation of *Src* was responsible for the acquired lapatinib resistance in two of seven isolated subclones in this *in vitro* model system. In addition, we further demonstrated that genetic or pharmacologic blockade of Src could restore ERBB2 inhibitor sensitivity in LR subclones with hyperactive Src. Although our data remain to be validated in patient samples, these data establish the role of oncogene *Src* as a pharmacologically tractable candidate mediator of acquired lapatinib resistance in ERBB2-expressed GE adenocarcinomas.

Recently, increased Src kinase activity was suggested as one of the resistance mechanisms to both trastuzumab and lapatinib in breast cancer cell lines [10], [39]. c-Src is a membrane-associated tyrosine kinase and cellular homologue of the oncogenic v-Src encoded by the chicken Rous sarcoma virus [40]. Src acts as a common signalling node by interacting with multiple receptor tyrosine kinases [10], [29], [41]. In breast cancer, Zhang et al demonstrated that increased Src kinase activity was responsible for both the *de novo* and acquired trastuzumab resistances. Separate investigators demonstrated that lapatinib-resistant SKBR3 breast cancer cells showed increased activity of Src kinases and persistent levels of activation of ERK 1/2 and AKT and that treatment with saracatinib reduced AKT and ERK 1/2 activity and restored lapatinib sensitivity [39]. A recent study by Han et al. similarly reported that increased Src activity was observed in the trastuzumab-resistant *ERBB2*-amplified GE cancer cell line, NCI-N87, and showed that trastuzumab and saracatinib synergistically inhibited the *in vitro* growth of both parental and trastuzumab-resistant NCI-N87 [18]. We also have generated lapatinib-resistant subclones from the NCI-N87 cell line, and performed similar sequencing in those subclones; however, we could not identify any similar *Src* mutations (data not shown). Ongoing efforts are trying to identify alternative etiology of resistance mechanisms in these lapatinib-resistant NCI-N87 subclones. Although the role of increased Src activity in the resistance of *ERBB2*-amplified breast cancer and GE cancer to ERBB2 inhibition has been documented, we reported here, for the first time, the activation of a spontaneous *Src* mutation after prolonged exposure to HER2 inhibitor could induce lapatinib resistance in *ERBB2*-amplified GE adenocarcinoma and present the first evidence of acquired mutation of *Src* as an etiology of resistance. While these data clearly demonstrate the capacity of activated Src to serve as a mediator of acquired resistance to ERBB2 inhibitor therapy in GE cancers, future studies will be required to query the presence of *Src* mutation or enhanced activity of this kinase in patient samples upon emergence of resistance.

Additionally, future studies will need to address potential differences between mechanisms of acquired resistance to small molecule compared to antibody ERBB2 inhibitors in GE adenocarcinomas. However, mechanisms for acquired resistance to trastuzumab have been reported to be similar to those to lapatinib in the breast cancer field. Mechanisms for *de novo* or acquired trastuzumab resistance included constitutive activation PI3-K pathway owing to *PTEN* deficiency or *PIK3CA* gene mutation [9], [42], the expression of truncated HER2 receptors [15], and overexpressions of other receptor tyrosine kinases which include EGFR, insulin-like growth factor-1 receptor, and hepatocyte growth factor receptor [43]–[45]. Mechanisms for lapatinib resistance were generally similar to those with the trastuzumab resistance [46]–[49].

Saracatinib, the c-Src/Abl dual targeting inhibitor, has shown only mild to moderate antitumor activity in phase I/II clinical trials [35]–[38]. However, these trials did not utilize genomic or biochemical biomarkers to guide enrolment nor did it investigate the situation of ERBB2 inhibitor insensitivity. Based upon the results presented in this study, further focused evaluation of Src activation, either due to mutation or other means of activation, should be considered on patient tumors following the acquisition of resistance to ERBB2-directed therapy for evidence. Such testing should evaluate for both activation due to mutation or from other mechanisms. Should such Src activation or *Src* mutation be identified in such tumor samples, the results from this report support subsequent efforts to perform clinical trials of a combination of ERBB2 inhibition and Src inhibition. Such therapy may be able to lead to meaningful improvements in outcomes for patients whose tumors utilize Src activation as a means of bypassing ERBB2 inhibition.

## Supporting Information

Figure S1 **Inferred copy-number plots for LR subclones.** Comparison of the copy-number profiles of the two *Src*-mutant LR subclones following normalization against the parental, lapatinib-sensitive OE19 cell line (x-axis: arbitrary chromosomal co-ordinates, color codes represent each chromosome in increasing order, y-axis: inferred copy-number).
(TIFF)

Figure S2 **Growth inhibition curves in the Two** ***Src*** **-mutant LR subclones after increasing dose of trastuzumab.** Values were presented as relative cellular viability relative to vehicle-treated controls with the mean ± S.E. of quadruplicate from a representative experiment. The *p* values calculated by two-way ANOVA were <0.0001 both in the comparisons of viabilities of OE19 *vs* LR2A and OE19 *vs* LR2B.
(TIFF)

Figure S3 **Growth inhibition curves in the parental OE19 and in the two** ***Src*** **-mutant LR subclones after increasing dose of saracatinib.** Values were presented as relative cellular viability relative to vehicle-treated controls with the mean ± S.E. of quadruplicate from a representative experiment. There was no statistical significance in terms of cell viability between cell lines.
(TIFF)

Table S1 Next-generation sequencing panel in 8 cell lines including parental OE19 and 7 lapatinib-resistant subclones.
(DOCX)
