# Supplementary figures and images for: Src Mutation Induces Acquired Lapatinib Resistance in ERBB2-Amplified Human Gastroesophageal Adenocarcinoma Models
Source: PLoS One. 2014 Oct 28;9(10):e109440. doi: 10.1371/journal.pone.0109440 (PMC4211679; doi:10.1371/journal.pone.0109440)

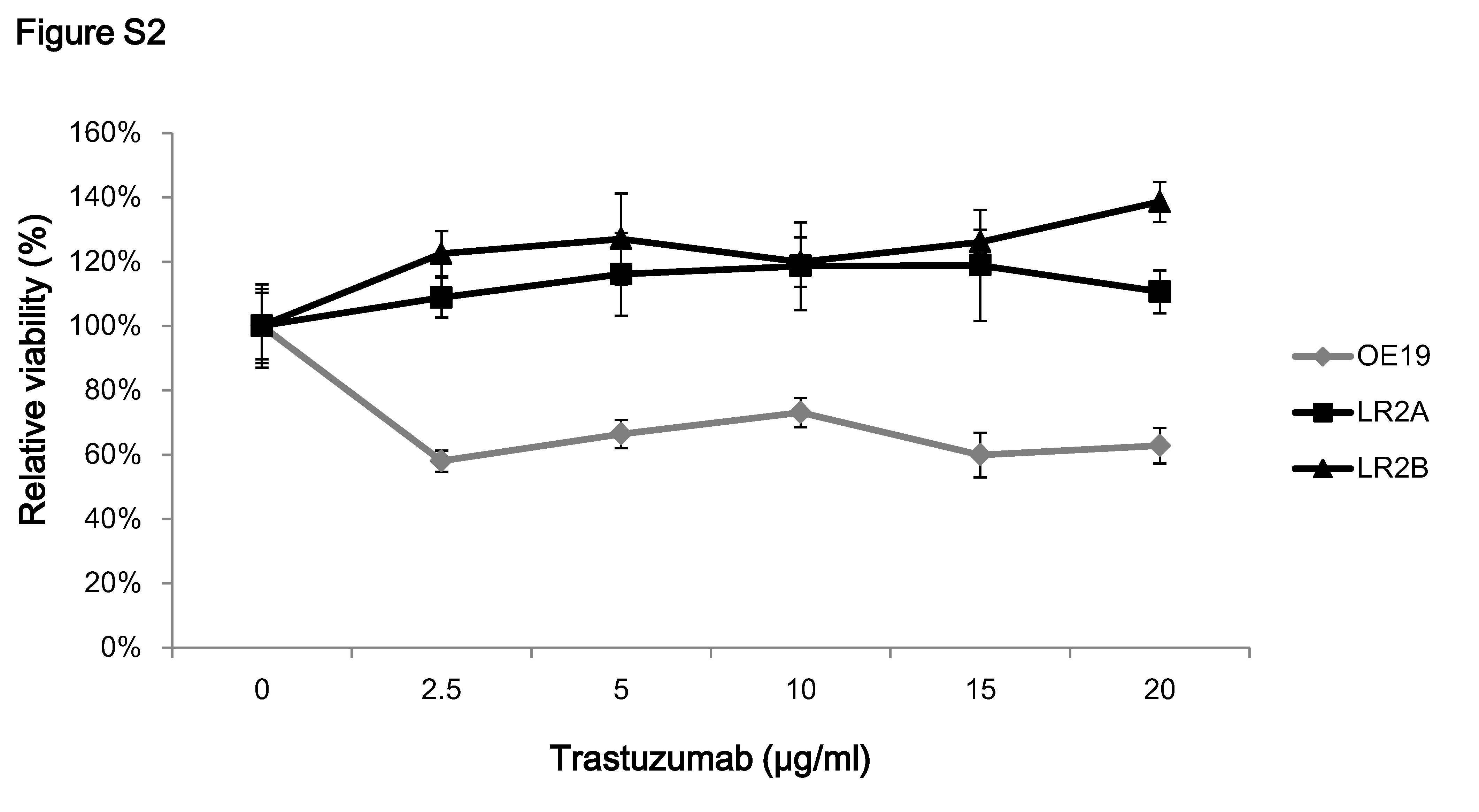

Supplement: Figure S2 — Growth inhibition curves in the Two Src -mutant LR subclones after increasing dose of trastuzumab. Values were presented as relative cellular viability relative to vehicle-treated controls with the mean ± S.E. of quadruplicate from a representative experiment. The p values calculated by two-way ANOVA were <0.0001 both in the comparisons of viabilities of OE19 vs LR2A and OE19 vs LR2B. (TIFF) [file pone.0109440.s002.tiff]

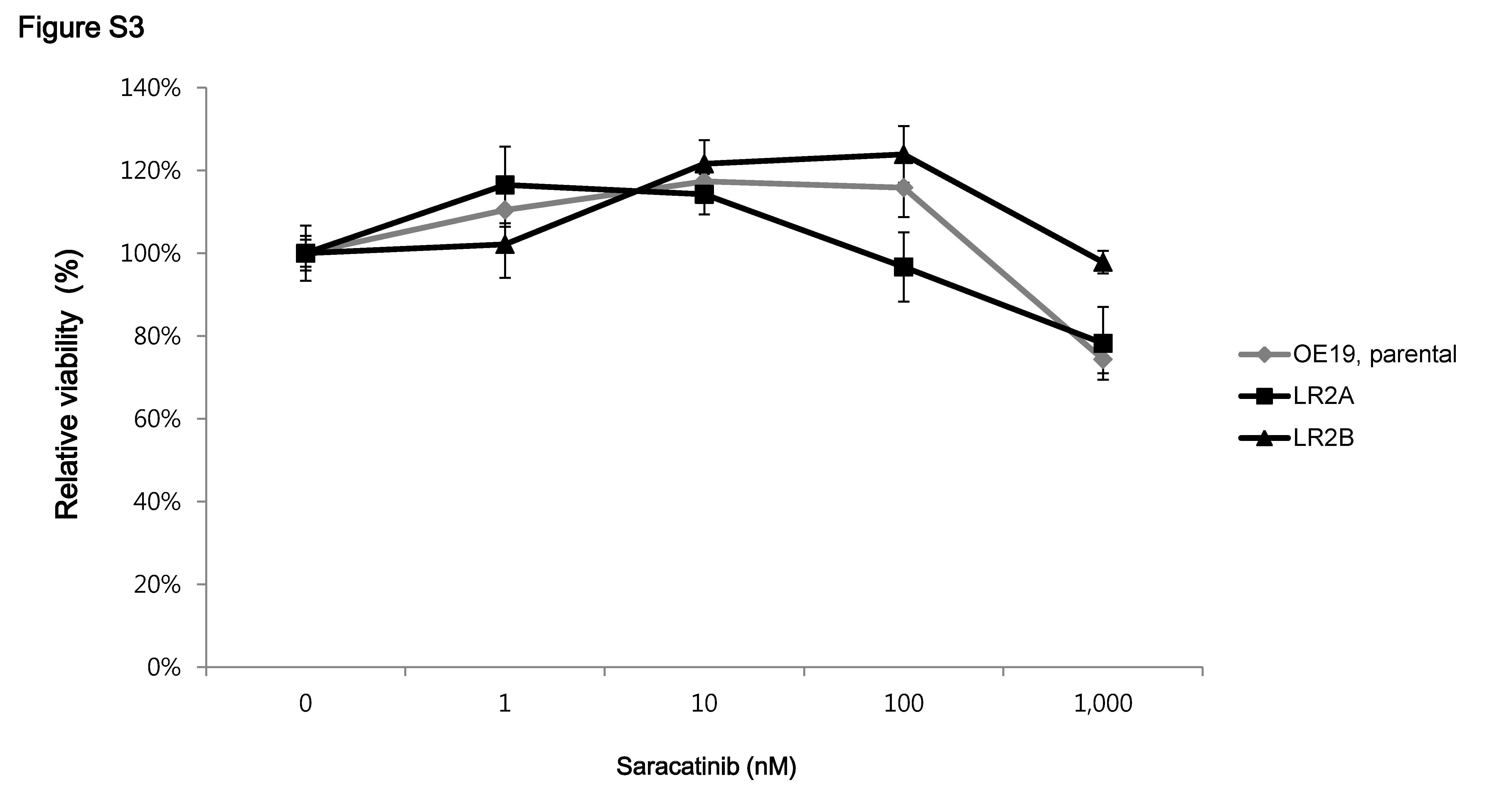

Supplement: Figure S3 — Growth inhibition curves in the parental OE19 and in the two Src -mutant LR subclones after increasing dose of saracatinib. Values were presented as relative cellular viability relative to vehicle-treated controls with the mean ± S.E. of quadruplicate from a representative experiment. There was no statistical significance in terms of cell viability between cell lines. (TIFF) [file pone.0109440.s003.tiff]
