# Supplementary material for: Src Mutation Induces Acquired Lapatinib Resistance in ERBB2-Amplified Human Gastroesophageal Adenocarcinoma Models
Source: PLoS One. 2014 Oct 28;9(10):e109440. doi: 10.1371/journal.pone.0109440 (PMC4211679; doi:10.1371/journal.pone.0109440)
Supplement: Table S1 — Next-generation sequencing panel in 8 cell lines including parental OE19 and 7 lapatinib-resistant subclones. (DOCX) [file pone.0109440.s004.docx]

**Table S1. Next-generation sequencing panel in8 cell lines including parental OE19 and 7 lapatinib-resistant subclones.**

| **Samples** | **No. of total reads** | **% Unique PF reads**  **aligned** | **% Selected Bases** | **Mean**  **Target coverage** | **% Targets**  **not covered** | **% Target bases**  **covered 30x** | **% Duplication** |
| --- | --- | --- | --- | --- | --- | --- | --- |
| OE19, parental | 43361466 | 96.8 | 60.1 | 249.5 | 0.3 | 97.2 | 65.7 |
| **LR2A** | 44254072 | 96.3 | 64.4 | 321.6 | 0.4 | 97.4 | 60.4 |
| **LR2B** | 49492118 | 96 | 62.5 | 317.9 | 0.4 | 97.7 | 63.5 |
| LR1 | 42221982 | 95.8 | 58.7 | 225.3 | 0.4 | 97 | 66.9 |
| LR3 | 41169108 | 96.5 | 58.5 | 217.4 | 0.4 | 96.9 | 67.6 |
| LR4 | 39893542 | 96.7 | 58.2 | 210.1 | 0.4 | 96.8 | 67.8 |
| LR5 | 38229836 | 96.8 | 61.9 | 240.9 | 0.4 | 96.9 | 63.9 |
| LR6 | 42318990 | 96.3 | 59.7 | 237.5 | 0.4 | 97 | 66.4 |
| Average | 42617639.3 | 96.4 | 60.5 | 252.5 | 0.4 | 97.1 | 65.3 |
